# St. George's Hospital

**Published:** 1914-01-31

**Authors:** 


### ST. GEORGE'S HOSPITAL.

### Princess Christian Resigns the Presidency.

The following official announcement was made on January 26: "Her Royal Highness the Princess Christian of Schleswig-Holstein has resigned the office of President of St. George's Hospital."

Princess Christian became President of St. George's Hospital on the accession of King George V., who resigned the presidency and became its patron. H.R.H. paid a formal visit to the hospital after accepting the office of President, when an address was presented to her, in the course of which it was stated that since St. George's Hospital was founded, in 1733, with the exception of a short period, it has been honoured by the reigning sovereign being its patron. Queen Victoria was patron and President of the hospital for nearly forty years, and then in the 'seventies became its President, and remained so until the time of her death. King Edward, on his accession in 1901, became patron, and, after a brief interval, the presidency was accepted by H.R.H. the Prince of Wales until his accession in 1910.

The members of the House Committee and staff of St. George's Hospital, in their address to Princess Christian in 1910, expressed their sense of the deep debt of gratitude which all sections of the community owed to H.R.H. for her unceasing interest in the welfare of the sick and needy, and more particularly for H.R.H.'s effort to raise the standard and welfare of trained nurses throughout the country. They also expressed great gratification at the appointment of H.R.H. Princess Victoria of Schleswig-Holstein as Vice-President of the hospital.

The chief matter of interest which has engaged the attention of the authorities of St. George's Hospital during the three and a-half years of Princess Christian's presidency has been the problem of how best to provide for the rebuilding of the hospital upon its present or a new site. That question is still unsolved, but substantial progress has been made in the direction of an amalgamation between St. George's and Westminster Hospitals, such amalgamation being contingent upon the sale of the sites on which these two hospitals stand.

In recent numbers of The Hospital we have given full particulars of the causes and circumstances attending the present crisis, which have culminated in the resignation of the President. Our efforts have been made in the direction of securing unanimity among the governors, and the meeting of the House Committee on January 28 gave evidence of the general desire exhibited by all parties to bring about this most desirable result.

### The President and the House Committee.

IMPORTANT CORRESPONDENCE.

*I. From H.R.H. Princess Christian, the President, To the House Committee of St. George's Hospital:—*

[Copy.]

Gentlemen,—I noted with gratitude the willingness of your Committee to assent to a private enquiry being made for me. I was anxious for this enquiry in order that I might become cognisant of all sides of the late differences of opinion, which up to the present time I am quite ignorant of. Certain governors, however, whilst not objecting to answering any questions put to them, distinctly state that they do *not* recognise any authority on my part, nor will they consent to abide by any decision arrived at as the result of such an enquiry.

This being the position, an enquiry would be useless, and it therefore falls to the ground.

I regret this extremely, as I had hoped to have been the means of bridging over the present difficulties.

It is with the deepest regret I find myself obliged to resign my position as President of the Hospital in which I have taken such a warm and real interest.—I remain, gentlemen, faithfully yours,

(Signed) Helena.

Cumberland Lodge, Windsor,
January 21, 1914.

*II. The Reply of the House Committee of St. George's Hospital to the President's letter of the 21st inst.:—*

The House Committee, after considering H.R.H. Princess Christian's letter, agreed to the following resolution, which was unanimously passed at the meeting on 28th inst.:—

"That the House Committee universally regret the resignation of H.R.H. Princess Christian, and earnestly request H.R.H. to reconsider her decision.

"They would respectfully request that she would be graciously pleased to receive a deputation from the House Committee to lay their views on this subject before her."

January 28, 1914.

St. George's Hospital,
Hyde Park Corner, S.W.,

It is universally desired that H.R.H. the President may be graciously pleased to receive the deputation asked for, because the House Committee desire that Princess Christian "may become cognisant of all sides of the late differences of opinion which up to the present time" H.R.H. declares herself to be "quite ignorant of." We hope that H.R.H. Princess Christian may consent to receive the proposed deputation, whatever her ultimate decision may be. There are good reasons why no final step shall, if possible, be called for in regard to H.R.H.'s letter of the 21st inst. earlier than the assembly of the June Court of Governors.

